# Clinical characteristics and treatments of cap polyposis: a single center case series

**DOI:** 10.1093/gastro/goad030

**Published:** 2023-05-30

**Authors:** Yanhua Zhou, Rui Cheng, Ningning Dong, Ying Meng, Chuntao Liu, Ye Zong

**Affiliations:** Department of Gastroenterology, Beijing Friendship Hospital, Capital Medical University, National Clinical Research Center for Digestive Disease, Beijing, P. R. China; Department of Gastroenterology, Beijing Friendship Hospital, Capital Medical University, National Clinical Research Center for Digestive Disease, Beijing, P. R. China; Department of Gastroenterology, Beijing Friendship Hospital, Capital Medical University, National Clinical Research Center for Digestive Disease, Beijing, P. R. China; Department of Gastroenterology, Beijing Friendship Hospital, Capital Medical University, National Clinical Research Center for Digestive Disease, Beijing, P. R. China; Department of Gastroenterology, Beijing Friendship Hospital, Capital Medical University, National Clinical Research Center for Digestive Disease, Beijing, P. R. China; Department of Gastroenterology, Beijing Friendship Hospital, Capital Medical University, National Clinical Research Center for Digestive Disease, Beijing, P. R. China

## Introduction

Cap polyposis is a rare benign disease of the digestive tract, characterized by inflammatory polyps with a cap of inflammatory granulation tissue. First described in 1985 by Williams *et al*. [1], cap polyposis is now still introduced in case reports. The clinical course and pathogenesis of this disease are yet to be elucidated and specific therapies have not been established.

## Patients and methods

In this study, we reported 14 patients with cap polyposis confirmed by endoscopy and pathology between January 2013 and December 2020 in Beijing Friendship Hospital, Capital Medical University (Beijing, China). The demographics, clinical manifestations, endoscopic and histopathological features, treatments, and outcomes of these cases were retrospectively analyzed.

## Results

### Clinical characteristics

There were 14 adult patients diagnosed with cap polyposis (Table 1), including 11 males and 3 females, with a median age of 58 years (range, 20–69 years). Common symptoms included hematochezia (7/14, 50%), diarrhea (6/14, 42.9%), and mucus stool (4/14, 28.6%). One patient (7.1%) had a history of ulcerative colitis and one patient (7.1%) had a history of rectal cancer treated by surgical operation. Four patients (28.6%) had no symptoms.

**Table 1.** Summary of 14 patients with cap polyposis

No.	Age	Gender	Clinical presentation	Localization	Number of lesions	Upper GI endoscopy	Hp infection	Hp eradiation	5-ASA	Other medical treatments	EMR	Surgery	Time of follow-up	Repeated colonoscopy	Outcome
1	37	Male	Diarrhea, hematochezia	Rectum	Three or more	No polyp	(+)	Yes	Topical	No	No	No	15 months	Not yet	Mild diarrhea and hematochezia
2	63	Male	Abdominal pain, diarrhea, hematochezia, mucus stool, rectal mucosal prolapse	From the transverse colon to the rectum	Three or more	No polyp	(–)	Yes	Oral and topical	Metronidazole, prednisone	Yes	No	12 months	Yes	Ineffective
3	66	Male	Constipation	Rectum	One	No polyp	(+)	Yes	No	No	Yes	No	6 months	Yes	Resolved
4	67	Female	No	Rectum	One	No polyp	(+)	Yes	No	NCo	Yes	No	13 months	Not yet	Resolved
5	34	Male	Abdominal pain, hematochezia, mucus stool, rectal mucosal prolapse	Rectum	Three or more	No polyp	(+)	Yes	No	No	Yes	No	6 years and 7 months	Yes	Resolved
6	23	Male	Hematochezia, rectal mucosal prolapse	Rectum	Three or more	No polyp	(–)	No	Topical	No	Yes	No	40 months	Yes	Small polyps on repeated colonoscopy
7	27	Male	Hematochezia	Rectum	Three or more	No polyp	(–)	No	Topical	Metronidazole	Yes	No	64 months	Yes	Small polyps on repeated colonoscopy
8	31	Female	Diarrhea	Sigmoid colon	One	No polyp	Not tested	No	No	No	Yes	No	20 months	Not yet	Resolved
9	63	Female	No	Rectum	Two	Not done	Not tested	No	No	No	Yes	No	54 months	Not yet	Resolved
10	67	Male	No	Rectum and sigmoid colon	Two	No polyp	Not tested	No	No	No	Yes	No	80 months	Yes	Resolved
11	69	Male	Abdominal pain, diarrhea, hematochezia, mucus stool, constipation	Rectum and sigmoid colon	Three or more	Not done	Not tested	No	Oral and topical	No	Yes	No	6 months	Yes	Small polyps on repeated colonoscopy
12	53	Male	Diarrhea, hematochezia, mucus stool	Rectum	Three or more	No polyp	(–)	No	No	No	Yes	No	33 months	Not yet	Resolved
13	63	Male	No	Rectal anastomosis	One	Not done	Not tested	No	No	No	No	Yes	39 months	Yes	Resolved
14	20	Male	Diarrhea	Rectum	Three or more	Not done	Not tested	No	No	No	No	Yes	15 months	Yes	Recurred; diarrhea

No., number; Hp, *Helicobacter pylori*; 5-ASA, 5-aminosalicylates; EMR, endoscopic mucosal resection; GI, gastrointestinal.

### Endoscopic features and histology

All patients underwent colonoscopy with findings of polyps. The endoscopic manifestations were polyps covered by a thick layer of white fibrinopurulent exudates. The polyps were most commonly located in the rectum only (10/14, 71.4%), in the sigmoid colon and the rectum (2/14, 14.3%), in the sigmoid colon only (1/14, 7.1%), and from the transverse colon to the rectum (1/14, 7.1%). The number of polyps ranged from one to several dozen. Four patients (28.6%) had only one polyp, two patients (14.3%) had two polyps, and eight patients (57.1%) had three or more polyps on colonoscopy. The Paris classification of the polyps was as follows: sessile (Is) in nine patients (64.3%), subpedunculated (Isp) in one patient (7.1%), pedunculated (Ip) in one patient (7.1%), Is and Isp in two patients (14.3%), and Isp and Ip in one patient (7.1%). A 23-year-old male patient with multiple Is rectal polyps was misdiagnosed with rectal cancer in another hospital but no malignant evidence was found pathologically.

Pathologic examination showed polypoid tissue exhibiting elongated, dilated, or tortuous hyperplastic colonic crypts in their central parts and covered in superficial regions by a “cap” of inflamed and ulcerated granulation tissue, fibrin, and inflammatory exudate.

Ten patients (71.4%) underwent upper gastrointestinal (GI) endoscopy and none of them had positive findings of cap polyposis. *Helicobacter pylori* (Hp) was tested in 8 of 14 patients (57.1%) via a ^13^C/^14^C-urea breath test (UBT). The positive rate of UBT was 50% (4/8).

### Management

The patients were treated with endoscopic mucosal resection (EMR), surgery, mesalazine, Hp eradication, and steroids alone or in combination. Eleven patients had EMR alone (four patients) or with medication (seven patients), two patients had surgical operation, and one patient was treated with medication (topical mesalazine and Hp eradication). All the four Hp-positive patients succeeded in eradiation.

### Follow-up results

All the patients were followed up. The median time of follow-up was 26.5 months (range, 6–79 months). EMR was effective for most patients: 10 patients were asymptomatic after EMR, of whom 6 patients significantly improved on colonoscopy (polyps of <0.6 cm that did not need to be treated were found in three patients and no cap polyposis occurred in the remaining three patients); 4 asymptomatic patients did not undergo a second colonoscopy after EMR. The patient with widespread colon involvement remained troubled by abdominal pain and diarrhea after oral and topical mesalazine, oral prednisone with a starting dose of 40 mg, Hp eradication despite a negative testing result and three procedures of EMR, and the repeated colonoscopy showed no improvement. One surgical patient had multiple rectal polyps on colonoscopy a year after surgery and the other surgical patient had no recurrence 3 years later. The patient who received medication remained with mild diarrhea and hematochezia.

## Discussion

In our series, the male gender predominated in cap polyposis and patients of a wide range of ages could be affected. Brunner *et al*. [2] found that cap polyposis could occur at any age and the youngest affected patient was aged 11 months. Cap polyposis predominantly affected the rectum and the sigmoid colon, occasionally other parts of the colon. The stomach could also be a target [3, 4]. However, none of the patients showed gastric involvement in our series.

As for the treatments, EMR was effective for most patients. Only in one patient with an extensive involvement of the colon did it turn out to be ineffective. Since it is less invasive, EMR should be recommended for most patients with rectosigmoid involvement, especially for those with few polyps. Endoscopic submucosal dissection (ESD) was also reported to be successful in an intractable cap polyposis with rectosigmoid involvement [5]. ESD might be a treatment option for cases that are refractory to conservative treatments.

Since Oiya *et al*. [3] reported a case of cap polyposis treated successfully by the eradication of Hp, Hp infection has been suggested to play a role in cap polyposis. However, in our study, none of the Hp-positive patients resolved after successful Hp eradication. Therefore, the role of Hp in cap polyposis is not certain and it remains to be elucidated who will benefit from Hp eradication.

Besides Hp eradication, several other medical treatments, including aminosalicylates, antibiotics, steroids, and infliximab [6, 7], have been reported with variable clinical outcomes. In our study, few patients used aminosalicylates, antibiotics, or steroids, and none of them showed a definite effect.

For the refractory patients, especially those with extensive involvement of the colon, surgical resection may be indicated. Two of our patients chose surgery, of whom one had recurrence a year after surgery and the other remained resolved >3 years after surgery.

From our study, the long-term prognosis was relatively good for most patients. However, there were still a small number of patients who were troubled by intractable symptoms, which greatly affected their quality of life.

To our knowledge, this is the largest case series in a single center. However, as a retrospective study, it still has some limitations. First, the number of cases is still too small to adequately evaluate the roles of different drug therapies including Hp eradication therapy. Second, for the refractory cases, we have not found any effective treatment besides surgery.

In conclusion, we reported 14 cases of benign cap polyposis and found that EMR has a definite treatment effect and should be the first choice if necessary. Surgical resection may be considered if medical and endoscopic therapy does not work.

## Authors’ Contributions

Study design was proposed by Y.H.Z. Data collection and analysis were accomplished by Y.H.Z. and R.C. Follow-up of patients was performed by N.D., Y.M., and C.L. The manuscript was drafted by Y.H.Z. and revised and edited by R.C. and Y.Z. All authors read and approved the final manuscript.

## References

[1] Williams GT , Bussey HR, Morson BC. Inflammatory “cap” polyps of the large intestine. Br J Surg 1985;72(suppl):S133.

[2] Brunner M , Agaimy A, Atreya R et al Cap polyposis in children: case report and literature review. Int J Colorectal Dis 2019;34:363–8. 30426196 10.1007/s00384-018-3192-6

[3] Oiya H , Okawa K, Aoki T et al Cap polyposis cured by Helicobacter pylori eradication therapy. J Gastroenterol 2002;37:463–6. 12108681 10.1007/s005350200067

[4] Al-Riyami AM , Lim BK, Peh WCG. Can the stomach be a target of cap polyposis? Endoscopy 2010;42:E124–5. 20405375 10.1055/s-0029-1214863

[5] Murata M , Sugimoto M, Ban H et al Cap polyposis refractory to Helicobacter pylori eradication treated with endoscopic submucosal dissection. World J Gastrointest Endosc 2017;9:529–34. 29085564 10.4253/wjge.v9.i10.529 PMC5648996

[6] Bookman ID , Redston MS, Greenberg GR. Successful treatment of cap polyposis with infliximab. Gastroenterology 2004;126:1868–71. 15188181 10.1053/j.gastro.2004.03.007

[7] Kim ES , Jeen YT, Keum B et al Remission of cap polyposis maintained for more than three years after infliximab treatment. Gut Liver 2009;3:325–8. 20431770 10.5009/gnl.2009.3.4.325 PMC2852720

